# Breathing Analysis Using Thermal and Depth Imaging Camera Video Records

**DOI:** 10.3390/s17061408

**Published:** 2017-06-16

**Authors:** Aleš Procházka, Hana Charvátová, Oldřich Vyšata, Jakub Kopal, Jonathon Chambers

**Affiliations:** 1 Department of Computing and Control Engineering, University of Chemistry and Technology in Prague, 166 28 Prague, Czech Republic; Vysatao@gmail.com (O.V.); Jakub.Kopal@vscht.cz (J.K.); 2 Faculty of Applied Informatics, Tomas Bata University in Zlín, 760 05 Zlín, Czech Republic; hcharvatova@email.cz; 3 Czech Institute of Informatics, Robotics and Cybernetics, Czech Technical University in Prague, 166 36 Prague, Czech Republic; 4 Faculty of Medicine in Hradec Králové, Department of Neurology, Charles University, 500 05 Hradec Kralove, Czech Republic; 5 School of Electrical and Electronic Engineering, Newcastle University, Newcastle upon Tyne, NE1 7RU, UK; Jonathon.Chambers@newcastle.ac.uk

**Keywords:** thermography, machine learning, facial temperature distribution, depth sensors, multimodal signals, breathing disorders detection

## Abstract

The paper is devoted to the study of facial region temperature changes using a simple thermal imaging camera and to the comparison of their time evolution with the pectoral area motion recorded by the MS Kinect depth sensor. The goal of this research is to propose the use of video records as alternative diagnostics of breathing disorders allowing their analysis in the home environment as well. The methods proposed include (i) specific image processing algorithms for detecting facial parts with periodic temperature changes; (ii) computational intelligence tools for analysing the associated videosequences; and (iii) digital filters and spectral estimation tools for processing the depth matrices. Machine learning applied to thermal imaging camera calibration allowed the recognition of its digital information with an accuracy close to 100% for the classification of individual temperature values. The proposed detection of breathing features was used for monitoring of physical activities by the home exercise bike. The results include a decrease of breathing temperature and its frequency after a load, with mean values −0.16 °C/min and −0.72 bpm respectively, for the given set of experiments. The proposed methods verify that thermal and depth cameras can be used as additional tools for multimodal detection of breathing patterns.

## 1. Introduction

The use of different sensors is essential for the study of many physiological and mental activities, neurological diseases [1,2] and motion and gait disorders [3,4]. The explanation of biomedical signals is also important for the development of assisted living technologies [5] and specific studies are related to polysomnography [6] and the study of many signals, including breathing and motion, as well as EEG and ECG signals.

Special attention is paid to temperature changes of facial parts affected by emotions, mental activities, or neurological disorders. The study of the temperature distribution over different parts of the face can be used in face and emotion detection [7,8,9,10,11,12], age recognition [13], motion [14], psychophysiology [15,16], neurology [17], and stress detection [18,19].

Noninvasive methods of breathing monitoring include electrical impedance tomography, respiratory inductance plethysmography [20,21], capnography and measurement of the tracheal sound, air capacity of the lungs or thoracic and abdominal circumference changes during respiration [22,23]. Thermal imaging can be used to measure both breathing rate and exhaled air temperature to provide useful information about the body load during physical activity and to study potential symptoms of certain respiratory diseases [24].

The respiratory rate is an important indicator [25] for monitoring of a person’s health. Some studies are devoted to sensing technologies in smart cities [26] that follow people’s vital signs without body instrumentation. These systems are often used for diagnosis of neurological disorders as well. A specific research is devoted to the respiration affect to cortical neuronal activity that modulates sensory, motor, emotional and cognitive processes [27].

The present paper is devoted to the noninvasive analysis of the breathing rate by facial temperature distribution using a thermal imaging camera [28,29] and by thorax movement monitoring recorded by the MS Kinect depth sensor. Data obtained from these instruments are then used for multimodal breathing analysis [30] and for monitoring of physical activities. Both the sequences of the thermal images and the depth matrices are acquired on the basis of contactless measurement.

A special attention is paid to the adaptive detection of facial thermographic regions. The present paper applies specific methods for their recognition allowing to detect the breathing rate or to recognize facial neurological disorders.

The proposed method of respiratory data processing is based on their statistical and numerical analysis using different functional transforms for the fast and robust estimation of desired features [20,23]. The respiratory rate estimation using chest motion analysis and facial thermographic data includes application of digital filtering and spectral analysis as well.

## 2. Methods

### 2.1. Data Acquisition

**Figure 1:**
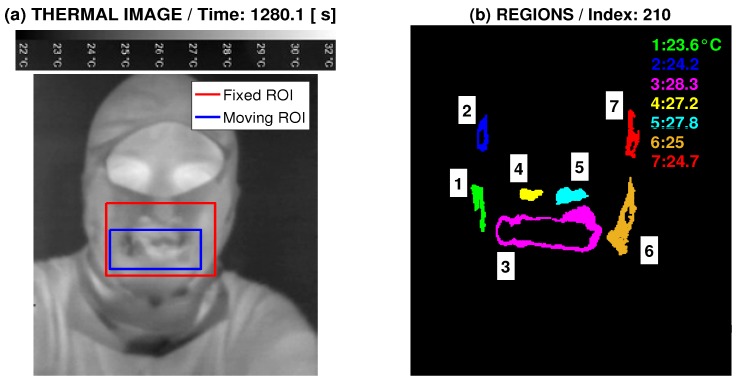
Data acquisition presenting (**a**) specification of the fixed and moving region of interest (ROI); (**b**) facial regions of different mean temperatures in the selected thermal image frame.

Figure 1 presents the general principle of the use of the thermal imaging camera [31] to detect temperature changes in the mouth area for the analysis of breathing. The calibration bar associated with each video frame is presented in the upper part of Figure 1a, showing the temperatures and associated image grey levels. Figure 1a presents the fixed ROI and the current position of the moving ROI as well. The recorded videosequence of temperature changes can then be used to analyse the time evolution of the temperature in the selected region of interest (ROI).

**Figure 2:**
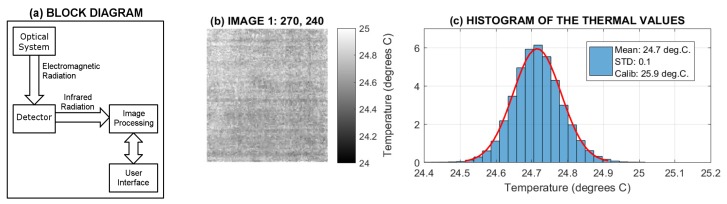
Thermal image analysis presenting (**a**) the block diagram of the thermal camera; (**b**) a selected image frame of a compact surface with equal temperature values; and (**c**) the distribution of values recorded by individual pixels.

The block diagram of the thermal imaging camera shown in Figure 2a presents its optical systems, radiation detector, and electronics for processing and presenting images. The lens projects thermal radiation to the radiation detector, which measures its intensity. This information is then digitized and transferred to the resulting thermogram. The basic parameters of the SEEK Compact thermal camera used in this study and listed in Table 1 include its optical resolution and temperature range. The sensor responds to long-wave infrared radiation with wavelengths between 7.5 and 14 μm.

**Figure 3:**
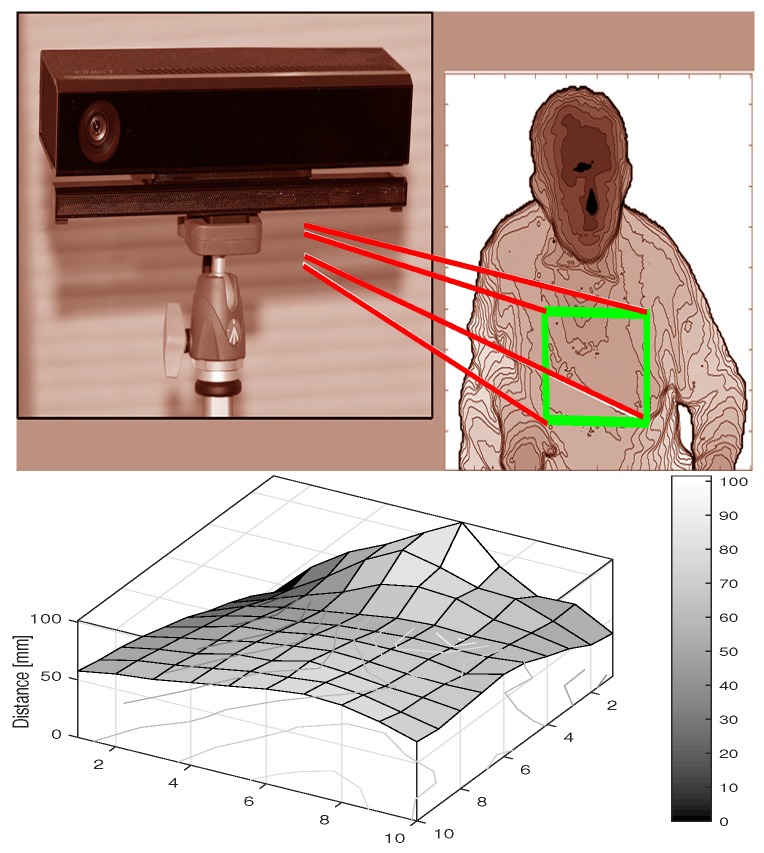
Principle of data acquisition by an MS Kinect depth sensor, presenting a selected depth frame with the regions of interest used to detect the chest movement.

An alternative approach to breathing data acquisition using an MS Kinect depth sensor is presented in Figure 3. For the selected thorax area, the depth sensing camera evaluates a matrix whose values indicate the distances of the individual pixels from the depth sensor. A videosequence of such frames can be used to determine the time evolution of chest movements in selected regions.

The range imaging methods used in depth sensors are based on the specific computational technologies that create the matrices whose elements carrying the information about the distance of the corresponding image component from the sensor [32,33]. The device features a sensor that is capable of capturing depth maps using the ‘Time of Flight’ technology [34]. The basic parameters of the MS Kinect used in this study are summarized in Table 1 as well.

### 2.2. Data Processing

The sequence of images recorded by the thermal camera were acquired with the changing temperature ranges associated with each videoframe as presented in Figure 1a. The adaptive recognition of these temperature ranges was performed by the two-layer neural network with the sigmoidal and softmax transfer functions [3] trained to recognize individual digits with the accuracy close to 100%. The classification model is able to detect the minimal and maximal temperature values in each thermographic frame and associated grey levels for the determination of the temperatures in each image.

The accuracy of the thermal camera was tested for a flat surface with equal temperature values and analysis of individual image frames as presented in Figure 2b,c. For applications requiring accurate temperature values the comparison with the calibrated thermometer is presented as well.

**Figure 4:**
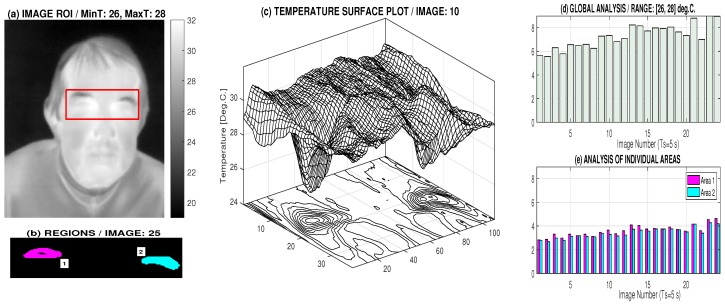
Thermal imaging camera accuracy analysis presenting (**a**) a selected thermal image with the region of interest (ROI) and the temperature bar; (**b**) areas specifying subregions with temperatures in the range of 26 °C and 28 °C; (**c**) the ROI temperature surface plot; (**d**) a global analysis of percentage of thermal pixels in the selected range of 26 °C and 28 °C; and (**e**) percentage values of thermal pixels in the selected range of 26 °C and 28 °C detected in two selected areas and their evolution for 24 images recorded for two minutes with a sampling period of 5 s.

Facial temperature values are useful for detection of neurological disorders and facial symmetry analysis. The time stability and precision of the thermal camera was tested for a sequence of images recorded with a sampling period of 5 s in the face area with a stable temperature distribution over a short period of time for a healthy individual. The area around the eyes illustrated in Figure 4a was selected and the area of the regions in the selected temperature range of 26–28 °C presented in Figure 4c was analysed. The results in Figure 4d,e enjoy a precision better than 7% related to the mean temperature, which is sufficient for the given case.

**Figure 5:**
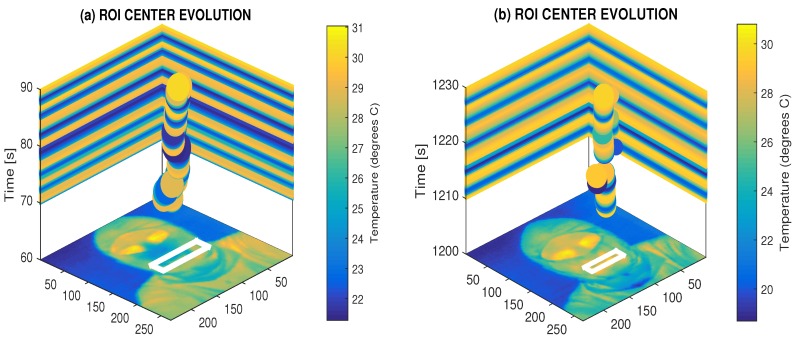
Principle of the use of the thermal imaging camera for breathing analysis, presenting the time evolution of the mean breathing temperature detected in the selected mouth region (**a**) during the physical exercise with the higher temperature and breathing frequency; (**b**) during the restful period with the lower temperature and breathing frequency.

The area (ROI) for the time evolution of temperature changes was specified empirically according to the first frame at first, as presented in Figure 5a. This fixed area assumed that the face maintained a stable position during the observation. To allow more flexible observations, the proposed algorithm includes the automatic detection of the area of interest using the following steps:

1. videorecording of the face area during a selected time range,
2. extraction of thermographic frames with the selected sampling frequency (of 10 Hz) and a given resolution,
3. automatic determination of temperature ranges in each thermographic frame and the adaptive calibration of each thermal image,
4. detection of the mouth area using the selected number of initial frames with the largest temperature changes and the adaptive update of this ROI for each subsequent thermal image,
5. evaluation of the mean temperature in the specified window of a changing position and size in each frame.

An example of the time evolution of the mean breathing temperature in the selected mouth region is presented in Figure 1a. The mean temperature in each video frame is associated with the grey level and the dot size in each time instant is in relation to the currently determined size of the mouth area.

An alternative analysis of breathing based upon thorax movement [1] was based on the mean value of the distance of the selected chest region from a MS Kinect depth sensor.

The analysis of multimodal records ${\left\{x\left(n\right)\right\}}_{n=0}^{N-1}$ of breathing obtained from the thermal imaging camera and depth sensors used similar signal processing methods. Their de-noising was performed by finite impulse response (FIR) filtering of a selected order, *M*, resulting in a new sequence ${\left\{y\left(n\right)\right\}}_{n=0}^{N-1}$ using the relation

(1) $$y\left(n\right)=\sum_{k=0}^{M-1}b\left(k\right)\;x\left(n-k\right)\;$$

with coefficients ${\left\{b\left(k\right)\right\}}_{k=0}^{M-1}$ defined to form a filter of the selected type and cutoff frequencies. In the case of breathing signals, a band pass filter was used to extract the frequency components in the range of 〈0.05,1.5〉 Hz.

The spectral components were then calculated by the discrete Fourier transform forming the sequence

(2) $$Y\left(k\right)=\sum_{n=0}^{N-1}y\left(n\right)\;\exp\left(-j\;k\;n\;\frac{2\;\pi }{N}\right)\;$$

for k=0,1,⋯,N-1 related to the frequency $f_{k}\;=\;\frac{k}{N}\times f_{s}$. For selected records 300 s long and a sampling frequency of $f_{s}=10$ Hz, resulting in each record being N=3000 samples long, the frequency resolution was $1/N\times f_{s}=0.0033$ Hz, which is sufficient for the given study.

## 3. Results

The detection of breathing features was verified during monitoring of physical activities by the home exercise bike. Each experiment was 40 min long and it included two periods of physical exercises followed by two restful periods with each of them 10 min long. The total number of 25 experiments was performed by one individual in similar home conditions.

Figure 5 presents the results of the adaptive detection of the mouth area and the evolution of the mean breathing temperature in the selected time range of 30 s with a sampling frequency of 10 Hz. The adaptive recognition of the temperature range in each frame is applied in this process as well.

**Figure 6:**
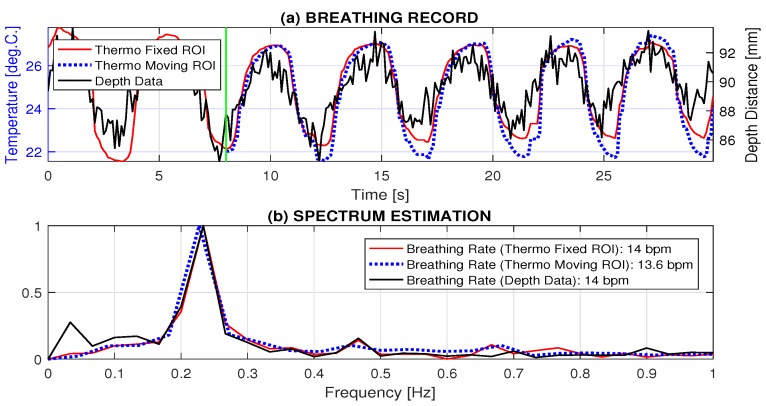
Data processing presenting (**a**) signals recorded by the thermal imaging camera and the MS Kinect depth sensor; and (**b**) detection of the breathing frequency from the fixed ROI, the moving ROI, and the MS Kinect depth sensor.

The resulting mean temperatures recorded by the thermal imaging camera evaluated from the fixed and the adaptively specified and moving temperature regions of interest are presented in Figure 6a. The moving ROI assumes its detection from temperature changes recorded during the selected number of previous frames. The history between 1 and 8 s long was selected (presented by the green vertical line in Figure 6a). As a result of the adaptive algorithm, the range of temperatures recorded by the moving ROI is larger, owing to the more precisely defined area of temperature changes following a possible slow movement of the head. The mean value of the distance of the chest area from the MS Kinect sensor recorded simultaneously is presented in Figure 6a as well.

Figure 6b presents the comparison of the breathing frequencies estimated from the spectral components evaluated for signals recorded both by the thermal imaging camera (using the fixed and moving ROI) and the MS Kinect depth sensor. All frequencies detected by this algorithm are the same for the given frequency resolution.

Table 2 presents the results of mean temperatures and temperature ranges evaluated in the fixed region of interest selected for the thermal imaging camera video records, as well as the adaptively changing positions and areas of this region evaluated by the proposed method. Table 2 presents further estimated breathing frequencies using the thermal imaging camera records. Since the records are 300 s long and the frame frequency is 10 Hz, all results are accurate within a frequency resolution of 0.0033 Hz (0.2 bpm). The same frequency was evaluated by the MS Kinect depth sensor which moreover provides information about thoracic and abdominal motion [1] during respiration.

**Figure 7:**
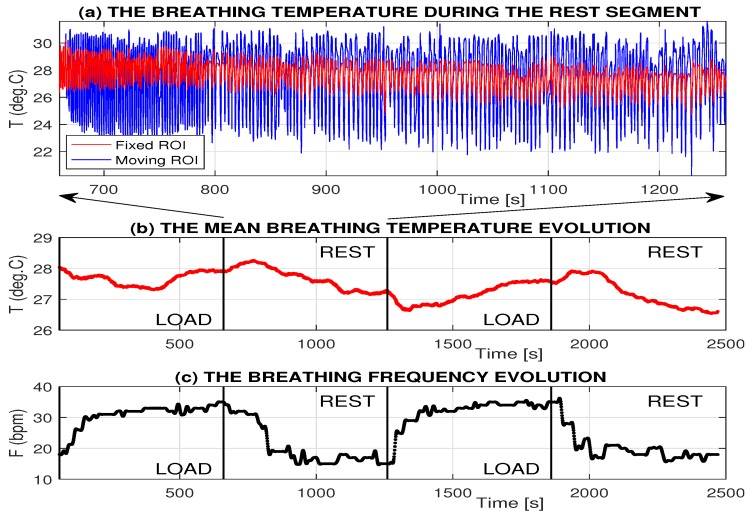
An example of records and results evaluated during two 10 min long segments of the physical exercises followed by two 10 min long resting time periods presenting (**a**) the time evolution of breathing temperatures; (**b**) the time evolution of the mean breathing temperature in a time window 60 s long; and (**c**) associated breathing frequency.

Both thermal imaging and motion data can be used for monitoring of the breathing rate during physical activity. The proposed method of adaptive specification of the breathing area and temperature range recognition was applied for the analysis of the evolution of the breathing features recorded during physical exercise and in the following resting time period. Figure 7 presents the breathing temperatures recorded by the thermal imaging camera in the selected time range and the corresponding evolution of mean temperatures and breathing frequency evaluated in time windows 60 s long. Figure 7a presents the evolution of mean temperatures in the fixed and moving mouth area showing wider temperature ranges for the moving ROI (specified in Table 2). The moving ROI position results in more robust detection of temperature changes allowing to follow motion of the head. Regression coefficients for the set of exercises 30 min long performed at room temperature and evaluated for the subsequent restful period of 7 min are summarized in Table 3. The resulting mean regression coefficients are −0.16 °C/min. and −0.72 bpm in the given case. These results correspond with the physiological explanation of breathing changes.

Table 4 presents mean delays of frequency and temperature changes related to the start of the physical exercise or restful period evaluated for 32 segments 10 min long. An example of a selected test is presented in Figure 7. Physiological needs cause faster change of the breathing frequency at the beginning of the physical activity. This increase of the air flow volume causes on the other hand a longer period of breath temperature change during segments with the physical exercise. Results of the delay of selected physiological functions related to changes of physical activities for cycling experiments [35] correspond to the tests specified above.

## 4. Conclusions

This paper proposes a method for the use of thermal and depth sensors to detect breathing features. The presentation includes a description of a machine learning method for the recognition of the temperature ranges as well as an adaptive specification of the mouth region using the sequence of thermographic images. The application is devoted to the study of selected physiological features evaluated during physical activities.

The results achieved show an example of simple sensors application for breathing analysis using simple thermal imaging cameras for the detection of temperature changes and MS Kinect depth sensors for the analysis of the motion in the chest area. The proposed method of thermographic regions detection allows both analysis of the breathing rate and the study of neurological problems in the facial area.

It is assumed that simple sensors can form an alternative tool for the detection of medical disorders, including sleep and breathing analysis in the home environment. 

## Figures and Tables

**Table 1:** Basic parameters of the thermal camera and MS Kinect sensors used for breathing analysis.

Thermo Camera Specifications			MS Kinect Specifications	
Feature	Description		Feature	Description
Thermal sensor resolution	206×156		RGB stream resolution	1920×1080
Detection distance	300 m		Depth stream resolution	512×424
Temperature range	−40–330 °C		Infrared stream resolution	512×424
Frame rate	<9 Hz		Depth range	0.4–4 m
Microbolometer	Vanadium Oxide		Frame rate	<30 Hz
Lens material	Chalcogenide			
Pixel pitch	12 μm			
Spectral range	7.5–14 μm			

**Table 2:** Mean temperatures (T), temperature ranges (R) and evaluated breathing frequencies (F) for fixed and changing regions of interest using thermal imaging camera records 5 min long acquired in the same restful periods of different physical tests.

Test	Fixed ROI				Moving ROI		
	T (°C)	R (°C)	F (bpm)		T (°C)	R (°C)	F (bpm)
1	26.49	3.49	14.79		27.19	10.07	14.79
2	26.26	3.17	15.61		27.02	9.12	15.61
3	26.79	5.66	15.41		27.09	10.61	15.41
4	27.33	4.31	15.82		27.47	11.38	15.82
5	26.14	4.00	16.23		26.95	9.44	16.23
6	27.55	4.20	14.58		27.32	9.07	14.58
7	27.54	4.13	16.64		27.40	9.98	16.64

**Table 3:** Regression coefficients and the mean squared errors S of the temperature and breathing frequency decrease during the time period of 7 min after the physical exercise 30 min long recorded by the thermal image camera.

Experiment	Temperature Evolution					Frequency Evolution			
	Reg. Coeff. [°C/min]	S [%]	Aver. Reg. Coeff.			Reg. Coeff. [bpm]	S [%]	Aver. Reg. Coeff.	
			Mean	STD				Mean	STD
1	−0.252	0.001				−0.513	0.401		
2	−0.182	0.001				−1.571	0.476		
3	−0.135	0.001	−0.162	0.059		−0.250	0.055	−0.720	0.619
4	−0.092	0.003				−0.117	0.508		
5	−0.148	0.001				−1.150	0.358		

**Table 4:** Mean delays of frequency and temperature changes related to the change of physical activity (physical exercise or restful period) for the set of 32 records 10 min long.

Breathing Feature	Segment	Mean Deleay (s)	STD
**Frequency**	**Load**	76	17
	**Rest**	98	47
**Temperature**	**Load**	188	59
	**Rest**	130	34

## Supplementary Materials

The following are available online at http://www.mdpi.com/1424-8220/17/6/1408/s1, S1: A video record of a selected set of thermal images showing the fixed and moving ROIs with facial regions of different mean temperatures and S2: a video record of selected depth frames acquired in the selected thorax area.
